# Generative artificial intelligence in secondary education: Applications and effects on students’ innovation skills and digital literacy

**DOI:** 10.1371/journal.pone.0323349

**Published:** 2025-05-09

**Authors:** Dang Wu, Jianyang Zhang

**Affiliations:** 1 School of Special Education, Handan University, Handan, China; 2 Faculty of Arts, University of Auckland, Auckland, New Zealand; National University of Lesotho, LESOTHO

## Abstract

As generative artificial intelligence (AI) rapidly transforms educational landscapes, understanding its impact on students’ core competencies has become increasingly critical for educators and policymakers. Despite growing integration of AI technologies in classrooms, there remains a significant knowledge gap regarding how these tools influence the development of essential 21st-century skills in secondary education contexts. This study addresses this gap by investigating the relationships between generative AI applications and two critical student outcomes: innovation capability and digital literacy. Through structural equation modeling analysis of data collected from 500 students across grades 7–12, the research reveals three key findings: Firstly, generative AI applications demonstrate a substantial positive effect on students’ innovation capability (β = 0.862, p < .001), enhancing critical thinking, creative problem-solving, and adaptive learning processes. Secondly, AI integration significantly improves digital literacy (β = 0.835, p < .001) by facilitating sophisticated information processing and active technological engagement. Thirdly, a strong bidirectional relationship exists between innovation capability and digital literacy (β = 0.791, p < .001), suggesting these competencies mutually reinforce each other in AI-enhanced learning environments. The model demonstrates robust explanatory power with excellent fit indices. By integrating the Technology Acceptance Model with Diffusion of Innovations theory, this study advances theoretical understanding of AI’s educational impact while providing practical guidelines for educators. The findings underscore the importance of strategic AI integration in educational curricula and suggest specific pathways for developing critical student competencies in the digital age.

## 1. Introduction

### 1.1. Research background

The emergence of generative artificial intelligence (AI) represents a transformative force in educational paradigms, fundamentally altering the dynamics of teaching and learning processes [1]. This technological evolution, occurring within the broader context of higher education disruption [2], emerges at a critical juncture where educational institutions confront unprecedented challenges in cultivating essential 21st-century competencies. Contemporary educational discourse increasingly emphasizes innovation capability and digital literacy as fundamental determinants of academic and professional success [3,4].

The technological landscape underpinning educational transformation has experienced exponential advancement, characterized by the complex interplay between organizational learning, distributed leadership, and digital technologies [5]. Within this context, generative AI—defined as AI systems capable of producing, manipulating, and analyzing content across multiple modalities—represents a fundamental paradigm shift in educational technology [6]. This shift manifests in the transformation of how students interact with information, construct knowledge, and develop critical competencies, necessitating systematic examination of educational innovations [7].

Innovation capability, conceptualized within the framework of 21st-century competencies, encompasses three primary dimensions: critical thinking, creative problem-solving, and adaptive learning capabilities [8]. Drawing from innovation diffusion theory, this capability represents the capacity to generate, evaluate, and implement novel solutions to complex problems [9]. The comparative analysis of international frameworks for 21st-century competencies reveals significant challenges in fostering these capabilities [10]. Traditional pedagogical approaches have encountered substantial limitations in addressing the demands of an increasingly technology-driven educational landscape [11] particularly within the digital networked world [12] While generative AI presents promising opportunities for enhancing innovation education, its effective integration requires careful consideration of both theoretical frameworks and pedagogical approaches suited for the 21st century [13,14] et al., 2016).

Digital literacy, evolving beyond basic technological competence, now encompasses a sophisticated complex of skills including critical information evaluation, digital content creation, and ethical technology utilization [15]. This evolution necessitates a pragmatic investigation into the nature and scope of digital literacy [16], aligning with the technology acceptance model that suggests effective technology integration depends on both perceived usefulness and ease of use [17]. In the context of generative AI, digital literacy has acquired new dimensions as students navigate an increasingly complex digital ecosystem where the boundaries between human-generated and AI-generated content become increasingly indistinct [18,19].

### 1.2. Problem statement

Despite growing recognition of generative AI’s transformative potential in education, significant gaps persist in our understanding of its influence on students’ development of innovation capability and digital literacy. Three critical research gaps warrant investigation: Firstly, the mechanisms through which AI tool utilization influences the development of innovation capability remain inadequately understood, particularly in basic education contexts. Secondly, the pathways by which generative AI shapes digital literacy acquisition lack systematic empirical examination. Thirdly, the potential synergistic relationship between innovation capability and digital literacy in AI-enhanced learning environments requires theoretical clarification and empirical validation [20]. These interrelated gaps significantly impede the effective integration of generative AI in educational practice.

### 1.3. Research purpose

This study aims to systematically investigate the impact of generative AI on students’ innovation capability and digital literacy development through a structural equation modeling approach. The research objectives progress from foundational analysis to theoretical synthesis:

Primary objective:

To establish a comprehensive theoretical model explaining the mechanisms through which generative AI influences student competency development.

Secondary objectives:

1. To delineate and empirically validate the pathways through which generative AI usage enhances students’ innovation capability development
2. To identify and analyze the specific mechanisms by which generative AI facilitates digital literacy acquisition
3. To examine the bidirectional relationship between innovation capability and digital literacy within AI-enhanced learning environments1.4 Research Hypotheses

This investigation proposes three interconnected hypotheses derived from the theoretical integration of innovation diffusion theory and the technology acceptance model. These hypotheses systematically address the research objectives by establishing testable propositions regarding the relationships between generative AI application, innovation capability, and digital literacy in educational contexts.

H1: Generative AI application has a significant positive effect on students’ innovation capability.

This hypothesis posits that students’ engagement with generative AI technologies facilitates enhanced innovation capability through facilitated cognitive processes. Innovation capability, conceptualized as a multidimensional construct encompassing critical thinking, creative problem-solving, and adaptive learning, is expected to develop through structured interaction with AI applications that provide personalized feedback, novel problem-solving approaches, and expanded informational resources.

H2: Generative AI application has a significant positive effect on students’ digital literacy.

This hypothesis proposes that engagement with generative AI technologies contributes substantively to the development of digital literacy competencies. Digital literacy, operationalized as the capacity to effectively evaluate, utilize, and create digital content, is anticipated to be enhanced through the sophisticated technological interactions necessitated by generative AI platforms, which require advanced information processing skills and critical digital engagement.

H3: There exists a significant positive relationship between students’ innovation capability and digital literacy in AI-enhanced learning environments.

This hypothesis suggests a bidirectional relationship between innovation capability and digital literacy, proposing that these competencies function synergistically within AI-enhanced educational contexts. The theoretical basis for this hypothesis lies in the conceptual overlap between innovation processes and digital competencies, where sophisticated information processing facilitates creative problem-solving, while innovative thinking enhances digital content creation and technological adaptation.

These hypotheses collectively establish a theoretical framework for examining the complex interrelationships between generative AI application and student competency development. They provide testable propositions that align with the research objectives and address the identified gaps in current understanding of AI’s educational impacts. The subsequent methodological approach is designed to empirically evaluate these hypotheses through robust structural equation modeling, enabling comprehensive assessment of both direct effects and potential synergistic relationships among the variables.

### 1.4. Research significance

This study contributes distinctively to the educational technology discourse through a tripartite framework of significance: theoretical advancement, methodological innovation, and practical utility. From a theoretical perspective, the research expands existing frameworks by synthesizing the Technology Acceptance Model with Diffusion of Innovations theory within the context of generative AI applications. This integration transcends conventional boundaries in educational technology research by articulating the mechanisms through which AI technologies influence cognitive processes in digital learning environments [21]. The theoretical framework developed herein addresses the fragmentation within educational technology discourse by providing a unified model that connects technological implementation with 21st-century competency development.

Methodologically, this research employs structural equation modeling to empirically validate theoretical propositions regarding generative AI’s impact on educational outcomes. This approach enables examination of complex interrelationships between latent constructs while accounting for measurement error—a sophisticated analytical strategy that strengthens causal inference within non-experimental designs. The model’s robust explanatory power for both innovation capability and digital literacy offers a significant advancement in quantifying technology-mediated educational outcomes, providing a methodological template for future investigations in this domain.

The practical significance emerges through the translation of theoretical insights into actionable implementation strategies for educational stakeholders. By delineating specific pathways through which generative AI influences student competencies, this research provides evidence-based guidance for curriculum development and technological integration. This practical utility extends beyond classroom application to inform policy formulation regarding digital technology implementation in educational institutions [15]. The research addresses the persistent gap between theoretical understanding and practical application in educational technology integration—a significant contribution given the rapidly evolving landscape of AI-enhanced learning environments.

By simultaneously advancing theoretical understanding, methodological approaches, and practical implementation strategies, this research responds directly to calls for more integrated perspectives on educational technology research [7]. The findings hold particular relevance in the current educational context where generative AI technologies are rapidly transforming pedagogical practices and learning modalities, offering a timely contribution to both the scholarly discourse and educational practice.

## 2. Literature review

### 2.1. Applications of generative AI in education

The integration of generative artificial intelligence in educational contexts represents a transformative development that fundamentally reconfigures traditional teaching and learning paradigms ([21,22]). This transformation, as Luckin et al. [14] cogently argue, necessitates a comprehensive reconceptualization of educational delivery mechanisms and pedagogical approaches. Through systematic analysis of existing research, scholars have identified multiple trajectories of AI implementation across various educational domains, with particular emphasis on the methodological rigor of integration strategies [23,24]. These investigations employ diverse methodological approaches, ranging from large-scale quantitative analyses to detailed qualitative case studies, thereby providing complementary perspectives on AI’s educational impact.

Zhai et al.‘s [25] comprehensive scoping review, utilizing a systematic coding methodology, identified key trends in AI educational applications. Their methodological framework, which emphasized construct validity and measurement reliability, revealed the emergence of sophisticated adaptive learning systems. This analysis extends earlier theoretical propositions by [26], who employed longitudinal analysis to predict AI’s evolutionary trajectory in education, demonstrating the progressive development from basic automation to complex personalized learning environments.

Contemporary research has particularly emphasized distance learning applications, as evidenced by Mijwil et al.‘s [27] mixed-methods investigation of AI-enhanced remote education. Their methodological approach, combining quantitative outcome measures with qualitative implementation analysis, revealed fundamental transformations in pedagogical interactions. This finding aligns with [28] theoretical framework regarding technology-mediated learning transformations. Williamson and Eynon’s [29] historical analysis further enriches this understanding by tracing the evolutionary trajectory of AI in education, while Khan [30] and Gravina [31] provide complementary perspectives on the practical implementation of intelligent tutoring systems and innovative educational approaches.

Critical perspectives have emerged through rigorous empirical investigation, notably in Selwyn’s [20] systematic analysis of automation assumptions in education. This work, complemented by Márquez and Henríquez’s [32] methodological framework for educational data mining, emphasizes the necessity of robust implementation strategies and continuous assessment protocols. The synthesis of these perspectives reveals the complex interplay between technological capability and pedagogical effectiveness.

### 2.2. Innovation ability research

The conceptualization and empirical measurement of innovation ability has emerged as a critical domain within educational research, particularly regarding technological integration. Beghetto and Kaufman [33] developed a comprehensive theoretical framework for innovation cultivation, employing construct validation studies to identify key environmental and psychological factors that facilitate innovative thinking development.

Significant methodological advances in innovation assessment have emerged through systematic investigation. Plucker and Makel’s [34] meta-analytic framework provides robust psychometric foundations for evaluating creative and innovative potential. This methodological rigor is further exemplified in Ritter and Mostert’s [35] experimental studies, which employed controlled trials to demonstrate the efficacy of cognitive-based innovation training, achieving strong internal validity through careful experimental design.

Environmental influences on innovation development have been systematically examined through Davies et al.‘s [36] comprehensive meta-analysis, complemented by [37] nuanced exploration of creativity in the digital age. Maley et al. [38] further extend this understanding through their analysis of creativity in educational contexts, while DeHaan [39] provides specific insights into fostering innovation within science education frameworks. Their methodological approach, incorporating both fixed and random effects models, identified statistically significant relationships between environmental characteristics and innovative output. These findings complement Sternberg’s [40] investment-based assessment framework, which provides psychometrically validated tools for measuring innovation capability.

### 2.3. Digital literacy research

Contemporary digital literacy research has evolved methodologically to encompass multidimensional competency assessment, as evidenced by the emergence of novel technological contexts such as the metaverse [41] The conceptual evolution of digital competence, thoroughly examined by Ilomäki et al. [42], has led to increasingly sophisticated theoretical frameworks that integrate multiple dimensions of technological capability. Spante et al.‘s [43] systematic review employed rigorous inclusion criteria and coding protocols to analyze the conceptual evolution of digital competence measurement. Their methodological framework reveals the complex interrelationships between various literacy components, demonstrating strong construct validity through factor analytic approaches.

Assessment methodologies have become increasingly sophisticated, as evidenced by Siddiq and Scherer’s [44] meta-analytic investigation of gender differences in ICT literacy. Their statistical approach, incorporating moderator analyses and publication bias assessment, provides robust evidence regarding demographic influences on digital competency development. This methodological rigor is further demonstrated in Hatlevik et al.’s [45] structural equation modeling analysis of self-efficacy and literacy outcomes.

The evolution of digital literacy frameworks demonstrates increasing methodological sophistication, particularly in [46] DIGCOMP model and its subsequent refinements [47]. These frameworks employ validated measurement instruments and demonstrate strong psychometric properties across diverse educational contexts. Law et al.‘s [48] global reference framework extends this methodological rigor to international assessment contexts. This international perspective is enriched by [49] systematic analysis of 21st-century digital skills and Ng’s [50] critical examination of digital natives’ learning patterns, providing crucial insights into the evolving nature of digital literacy in contemporary educational environments.

### 2.4. Theoretical foundation and integration

The theoretical foundation of this research domain integrates three primary frameworks, each contributing distinct yet interconnected perspectives on technological innovation in education. The Technology Acceptance Model [50] provides empirically validated constructs for understanding technology adoption processes, particularly through perceived usefulness and ease of use as determinants of user attitudes and behavioral intentions toward emerging technologies. Innovation Diffusion Theory [51] offers complementary insights into implementation dynamics, elucidating how innovations propagate through social systems, gain acceptance, and ultimately transform practice through structured adoption stages and innovation characteristics. Digital Literacy Development Theory [15] contributes the third critical perspective, conceptualizing the multidimensional nature of digital competencies as they progress from technical operational skills to sophisticated information evaluation and creative content generation capabilities. These three theoretical frameworks synthesize to create a comprehensive model capable of capturing the complex interplay between technological affordances, adoption processes, and competency development in educational contexts.

This integrated theoretical framework reveals several key mechanisms through which generative AI influences student development:

Technology Acceptance Pathway: TAM-based research [52,53] et al., 2019) demonstrates how perceived usefulness and ease of use mediate the relationship between AI implementation and student engagement. The cognitive and affective dimensions of technology acceptance significantly influence both the initial adoption of generative AI tools and their sustained utilization in learning contexts, ultimately shaping the potential developmental outcomes.

Innovation Diffusion Process: DOI theory ([54,55]) explains the temporal and social aspects of AI adoption, particularly regarding how innovative capabilities develop through technology exposure. The theory provides a structured framework for understanding the progressive stages through which educational stakeholders incorporate generative AI into instructional practice and how these technologies diffuse through educational communities with varying rates and patterns.

Digital Competency Development: Theoretical work by [56,57] provides frameworks for understanding how AI interaction enhances digital literacy through active engagement with sophisticated technological systems. This perspective emphasizes the recursive relationship between technological interaction and competency development, where increasingly complex digital environments facilitate the progressive elaboration of multidimensional literacy capabilities through structured engagement with advanced technological systems.

The synthesis of these three theoretical perspectives—technology acceptance, innovation diffusion, and digital literacy development—establishes a comprehensive framework for examining how generative AI applications influence educational outcomes. This integrated approach transcends the limitations of singular theoretical perspectives, enabling more nuanced analysis of the complex, multidimensional processes through which technological innovation transforms educational practice and student development. The theoretical integration accommodates both micro-level cognitive processes and macro-level organizational dynamics, providing a robust analytical framework for examining the transformation of educational environments through generative AI implementation.

### 2.5. Research commentary

Critical analysis of the existing literature reveals several methodological and theoretical implications. While individual research streams demonstrate robust empirical foundations, the integration of AI applications, innovation ability, and digital literacy requires more sophisticated theoretical modeling. Current methodological approaches, though rigorous within their domains, may not fully capture the complex interactions between these components.

Future research directions should emphasize:

1. Development of integrated measurement models that simultaneously assess AI usage patterns, innovation capabilities, and digital literacy outcomes
2. Implementation of longitudinal designs with stronger internal validity for causal inference
3. Investigation of cultural and contextual moderators in the AI-capability development relationship

Examination of pedagogical mediators in the technology-outcome relationshipThis systematic analysis demonstrates the need for more sophisticated methodological approaches that can capture the dynamic interplay between technological tools, student capabilities, and educational outcomes. Such methodological advancement would enhance both theoretical understanding and practical implementation strategies.

## 3. Research methodology

### 3.1. Research design

The selection of structural equation modeling (SEM) as the primary analytical framework is particularly appropriate given the latent nature of the core constructs and the hypothesized complex interdependencies among variables. This methodological choice enables simultaneous estimation of multiple dependency relationships while incorporating measurement error - capabilities essential for examining the multifaceted relationships between generative AI application, innovation ability, and digital literacy. The cross-sectional design, while acknowledging temporal limitations, provides a robust framework for examining the structural relationships among these constructs at a critical juncture in educational technology implementation.

The research framework operationalizes three primary constructs through a carefully constructed measurement model. Generative AI application, conceptualized as the exogenous variable, encompasses both behavioral and attitudinal dimensions of technology engagement. Innovation ability and digital literacy, positioned as endogenous variables, are theoretically specified to capture the multidimensional nature of these educational outcomes. This framework allows for rigorous examination of both direct effects and potential mediating relationships, while controlling for measurement error through latent variable modeling.

### 3.2. Research subjects

The sampling framework employed a multistage stratified random sampling procedure to ensure comprehensive representation across educational contexts. Initial stratification criteria included grade level (junior and senior secondary), school type (public and private institutions), and geographic location (urban and suburban areas), with probability proportional to size sampling within each stratum. This approach optimizes both the efficiency and representativeness of the sample, while maintaining statistical precision for subgroup analyses.

Sample size determination followed a comprehensive power analysis protocol, incorporating both statistical power requirements for SEM (minimum sample size for desired power of.80 at α = .05) and practical considerations regarding model complexity (number of parameters to be estimated). The target sample size of 500 was established to maintain a minimum ratio of 10 observations per parameter, ensuring stable parameter estimates and adequate statistical power for model testing. The realized sample demonstrated satisfactory demographic balance, with proportional representation across grade levels (grades 7–12: 16.5% ± 1.2% per grade) and gender distribution (female: 51.3%, male: 48.7%).

### 3.3. Variable measurement

The measurement framework was developed through a systematic scale construction process integrating theoretical foundations with empirical validation. The instrument development procedure followed a four-phase protocol: (1) theoretical domain specification and item generation, (2) expert review and content validation, (3) cognitive interviewing and item refinement, and (4) pilot testing and psychometric evaluation.

Initial item pools were generated through comprehensive literature review and expert consultation, with particular attention to content validity and construct representation. The expert review panel, comprising six specialists in educational technology, innovation research, and psychometrics, evaluated item relevance, clarity, and construct alignment using a standardized rating protocol. Items achieving an Item-Level Content Validity Index (I-CVI) ≥.83 were retained for further evaluation.

Cognitive interviews with target population representatives (n = 12) enabled assessment of item comprehension and response processes. This phase resulted in refinement of item wording and response category optimization. The pilot testing phase (n = 50) provided preliminary evidence of scale reliability (Cronbach’s α ranging from.82 to.91) and construct validity through exploratory factor analysis.

The final instrument comprises:

The generative AI application scale integrates behavioral indicators of technology use with attitudinal measures, employing precisely calibrated five-point Likert-type items. The innovation ability assessment framework operationalizes seven theoretically-derived dimensions through behaviorally-anchored rating scales. The digital literacy measure synthesizes established competency frameworks into a comprehensive assessment tool, with items calibrated to capture both basic and advanced digital capabilities.

### 3.4. Data collection

Data collection procedures followed a standardized protocol to ensure measurement consistency and data quality. The implementation framework specified uniform testing conditions, administrator training requirements, and quality control measures. Survey administration occurred during regular academic sessions over a four-week period, with standardized instructions and response time allocations.

Quality assurance measures included:

1. Standardized administrator training and certification
2. Real-time response quality monitoring
3. Systematic response pattern analysis
4. Structured follow-up procedures for incomplete responses

Data cleaning protocols employed a systematic approach to missing data analysis, incorporating both pattern analysis and imputation procedures where appropriate. Cases with more than 10% missing data were excluded from the analysis, while remaining missing values were addressed through multiple imputation procedures to maintain sample integrity while minimizing potential bias.

### 3.5. Data analysis

The analytical framework employed a systematic two-phase approach to model evaluation. The measurement model assessment phase began with preliminary data screening, including evaluation of univariate and multivariate normality, outlier detection, and initial assessment of variable relationships. Confirmation of the measurement model proceeded through confirmatory factor analysis, with thorough examination of factor structure, convergent validity, discriminant validity, and reliability assessment to ensure measurement integrity.

The structural model evaluation phase utilized robust maximum likelihood estimation to account for potential non-normality in the data distribution. Model fit assessment incorporated multiple complementary indices evaluated against established thresholds: χ²/df ratio (acceptable fit < 3.0), RMSEA (good fit < .06, acceptable < .08), CFI and TLI (good fit > .95, acceptable > .90), and SRMR (good fit < .05, acceptable < .08). These thresholds reflect contemporary methodological consensus in structural equation modeling research and were selected based on their demonstrated efficacy in educational technology contexts [58,59], 2016).

Reliability evaluation employed similarly rigorous threshold criteria including internal consistency (Cronbach’s α ≥ .80), composite reliability (CR ≥ .80), and Average Variance Extracted (AVE > .50), following established guidelines in psychometric literature ([60,61]. Hypothesis testing incorporated examination of direct effects through standardized path coefficients, with significance assessed through both parametric tests and bootstrap-derived confidence intervals. Effect size estimation employed standardized solutions and explained variance proportions to evaluate practical significance.

Model modification decisions followed a theoretically-guided empirical approach, with modifications considered only when theoretically justified and resulting in substantial improvement in model fit. Potential multicollinearity concerns were addressed through composite reliability analysis and discriminant validity assessment using the Heterotrait-Monotrait ratio criterion, supplementing the traditional Fornell-Larcker approach to ensure robust construct differentiation [62].

All analyses were conducted using Mplus version 8.3 for structural equation modeling and SPSS version 28.0 for preliminary data screening and descriptive analyses.

### 3.6. Ethical considerations

This empirical investigation was conducted under the institutional oversight and ethical approval of the Faculty of Social Sciences Ethics Committee at the University of Auckland (Reference Number: UAHPEC/2024/125), with the data collection phase implemented between March 15, 2024, and May 31, 2024. All research procedures were executed in strict accordance with the university’s human participant research guidelines and the New Zealand National Ethics Advisory Committee requirements for educational research involving minors.

Written informed consent was obtained through a rigorous two-tier process, differentiated by participant age cohorts. For participants aged 16 and above, standard informed consent procedures were implemented. For participants under 16 years of age, a comprehensive dual-consent protocol was employed, requiring written authorization from both the student participants and their parents or legal guardians. The consent documentation encompassed detailed explications of: (1) the study’s theoretical framework and empirical objectives, (2) methodological procedures and participant obligations, (3) potential benefits and anticipated risks, (4) data management protocols and privacy safeguards, and (5) participants’ unconditional right to withdraw from the study. Age-appropriate information sheets were developed through cognitive interviewing procedures to ensure comprehensibility for adolescent participants, while maintaining methodological rigor in content delivery.

The voluntary nature of participation was emphasized throughout the recruitment and data collection phases, with explicit provisions for data withdrawal implemented up until the commencement of the analytical phase (June 1, 2024). Data confidentiality was preserved through systematic anonymization protocols, employing algorithmic identifiers to replace personally identifiable information during data processing. In compliance with institutional data retention policies and international research standards, all research data will be maintained in encrypted digital storage for a six-year period following study completion, after which it will undergo secure digital destruction protocols in accordance with the University of Auckland’s data management guidelines and New Zealand privacy legislation.

## 4. Results

### 4.1. Descriptive statistics

Initial examination of measurement dimensions revealed systematic patterns across key study variables. As shown in Table 1, the assessment of AI application dimensions demonstrated consistent central tendencies (M = 2.96–3.05) with moderate dispersion parameters (SD = 0.70–0.77), suggesting stable measurement properties. The AI Use Breadth dimension exhibited marginally higher mean scores (M = 3.05, SD = 0.73), indicating more extensive engagement patterns relative to other application domains.

**Table 1:** Descriptive Statistics and Correlations of Key Variables.

Variables	Mean	SD	1	2	3	4	5	6
1. AI Use Frequency (AI1)	2.98	0.7	1					
2. AI Use Depth (AI2)	2.97	0.73	.76**	1				
3. AI Use Breadth (AI3)	3.05	0.73	.78**	.81**	1			
4. AI Use Effect (AI4)	2.96	0.75	.77**	.80**	.81**	1		
5. AI Use Attitude (AI5)	2.98	0.77	.80**	.83**	.85**	.84**	1	
6. Innovation Capability	4.02	0.72	.74**	.77**	.78**	.78**	.81**	1
7. Digital Literacy	3.58	0.68	.72**	.74**	.76**	.75**	.78**	.79**

Note: ** p < .01.

Analysis of sample composition revealed systematic stratification across demographic parameters that align with the study’s methodological framework (see Table 2). The proportional distribution across educational levels (16.4–16.8% per grade) and balanced gender representation (51.4% female) enhanced sample characteristics. Notable patterns emerged in prior AI experience, with a theoretically consistent distribution demonstrating modal concentration in basic proficiency (41.0%), complemented by balanced representation in preliminary (25.0%) and intermediate (24.0%) competency levels.

**Table 2:** Demographic Characteristics of Research Participants (N = 500).

Characteristics	Category	Frequency	Percentage
Gender	Female	257	51.40%
	Male	243	48.60%
Grade Level	Grade 7	83	16.60%
	Grade 8	82	16.40%
	Grade 9	84	16.80%
	Grade 10	83	16.60%
	Grade 11	84	16.80%
	Grade 12	84	16.80%
School Type	Public	300	60.00%
	Private	200	40.00%
Geographic Location	Urban	285	57.00%
	Suburban	215	43.00%
Prior AI Experience	None	125	25.00%
	Basic	205	41.00%
	Intermediate	120	24.00%
	Advanced	50	10.00%

### 4.2. Measurement model assessment

Rigorous psychometric evaluation revealed robust reliability and validity characteristics across measurement instruments. As evidenced in Table 3, internal consistency indices exceeded conventional thresholds (Cronbach’s α = 0.873–0.912), while composite reliability coefficients (CR = 0.883–0.912) provided additional support for scale consistency. Convergent validity assessment through Average Variance Extracted (AVE = 0.798–0.837) demonstrated strong indicator cohesion, substantially exceeding established criteria.

**Table 3:** Scale Reliability and Validity Analysis.

Constructs	Cronbach’s α	CR	AVE	MSV	ASV
AI Application	0.873	0.897	0.816	0.743	0.687
Innovation Capability	0.912	0.912	0.837	0.743	0.701
Digital Literacy	0.883	0.883	0.798	0.626	0.584

Note: CR = Composite Reliability; AVE = Average Variance Extracted; MSV = Maximum Shared Variance; ASV = Average Shared Variance.

Further decomposition of reliability assessments at the dimensional level revealed robust psychometric properties across the five constituent dimensions of AI application. The AI Use Frequency dimension demonstrated strong internal consistency (Cronbach’s α = 0.889), indicating coherent measurement of interaction regularity. The AI Use Depth dimension exhibited similarly robust reliability coefficients (Cronbach’s α = 0.902), reflecting consistency in respondents’ articulation of engagement intensity with generative AI technologies. Particularly noteworthy was the internal consistency of the AI Use Breadth dimension (Cronbach’s α = 0.921), suggesting stability in participants’ responses across diverse application contexts. The AI Use Effect dimension (Cronbach’s α = 0.896) and AI Use Attitude dimension (Cronbach’s α = 0.914) both yielded theoretically congruent coefficients that substantially exceeded conventional thresholds. These dimensional reliability indices surpass established criteria in information systems research (Nunnally & Bernstein, 1994), providing empirical support for measurement stability. Complementary composite reliability coefficients for each dimension (CR = 0.891–0.927) further corroborated measurement robustness, demonstrating that these dimensions effectively represent their theoretical constructs while maintaining sufficient distinctiveness to capture the multidimensional nature of AI application experiences. This dimensional reliability analysis enhances confidence in both the overall measurement model and the structural relationships derived from it, supporting the validity of subsequent path analyses that treat AI application as a higher-order latent construct.

The substantial correlations among AI application dimensions (r = .76-.85) necessitated methodological attention to address multicollinearity concerns. Variance Inflation Factor analysis yielded values (VIF = 3.42–4.18) below the conservative threshold of 5.0 (Hair et al., 2019), indicating acceptable multicollinearity levels. Additionally, Heterotrait-Monotrait ratio assessment (Henseler et al., 2015) produced indices (.78-.86) below the.90 threshold, confirming adequate construct differentiation despite high correlations. Model comparison tests demonstrated superior fit for the multidimensional structure (Δχ² = 127.46, Δdf = 7, p < .001), while alternative specifications using AI application as a second-order construct yielded nearly identical path coefficients (Δβ < .03), demonstrating result robustness across modeling approaches.

The Fornell-Larcker criterion assessment (see Table 4) provided empirical support for construct distinctiveness, with AVE square root values (0.893–0.915) systematically exceeding inter-construct correlations. Supplementary analysis of Maximum Shared Variance and Average Shared Variance indices corroborated discriminant validity findings.

**Table 4:** Discriminant Validity Assessment (Fornell-Larcker Criterion).

Constructs	1	2	3
1. AI Application	0.903		
2. Innovation Capability	0.862	0.915	
3. Digital Literacy	0.835	0.791	0.893

Note: Bold diagonal values are the square root of AVE; Off-diagonal values are correlations between constructs.

Factor structure analysis revealed robust measurement properties across all indicators (see Table 5). Standardized loadings demonstrated strong construct representation (λ = 0.744–0.948), with particularly robust manifestation in AI application indicators (λ = 0.858–0.934). The statistical significance of factor loadings (t = 19.612–78.466, p < .001) provided additional support for measurement model validity.

**Table 5:** Factor Loadings.

Constructs and Items	Factor Loading	SE	t-value
AI Application (AI)			
AI1: Use Frequency	0.858	0.031	27.717
AI2: Use Depth	0.89	0.025	35.23
AI3: Use Breadth	0.906	0.023	40.221
AI4: Use Effect	0.898	0.022	41.588
AI5: Use Attitude	0.934	0.015	61.902
Innovation Capability			
Innovation Thinking	0.744	0.038	19.612
Problem Solving	0.948	0.012	78.466
Creative Expression	0.919	0.016	55.893
Innovation Practice	0.917	0.019	48.273
Innovation Consciousness	0.943	0.014	69.13
Innovation Attitude	0.928	0.017	55.491
Innovation Behavior	0.932	0.018	50.959
Digital Literacy			
Information Acquisition	0.858	0.026	32.832
Information Evaluation	0.907	0.018	51.551
Information Application	0.878	0.026	34.194
Digital Collaboration	0.902	0.02	45.252
Digital Creation	0.901	0.022	40.594
Digital Ethics	0.898	0.023	39.729
Digital Security	0.91	0.019	47.181

Comprehensive fit assessment revealed satisfactory model characteristics across multiple indices (see Table 6). The ratio of chi-square to degrees of freedom (χ²/df = 1.704) indicated appropriate model parsimony, while the Root Mean Square Error of Approximation (RMSEA = 0.075) suggested acceptable approximation error. Incremental fit indices (CFI = 0.963, TLI = 0.952) and residual assessment (SRMR = 0.042) collectively supported measurement model adequacy.

**Table 6:** Measurement Model Fit Indices.

Fit Index	Value	Acceptable Threshold
χ²/df	1.704	< 3.0
RMSEA	0.075	< 0.08
CFI	0.963	> 0.95
TLI	0.952	> 0.95
SRMR	0.042	< 0.06

### 4.3. Structural model analysis

Path analysis revealed theoretically consistent structural relationships (see Table 7). AI application demonstrated substantial positive effects on both innovation capability (β = 0.862, t = 32.590, p < .001) and digital literacy (β = 0.835, t = 25.941, p < .001). The hypothesized bidirectional relationship between innovation capability and digital literacy showed significant positive association (β = 0.791, t = 17.486, p < .001).

**Table 7:** Path Analysis Results and Model Effects.

Path	Standardized Coefficient (β)	SE	t-value	P-value
AI Application to Innovation Capability	0.862	0.026	32.59	<.001
AI Application to Digital Literacy	0.835	0.032	25.941	<.001
Innovation Capability and Digital Literacy	0.791	0.045	17.486	<.001

R² Values:

•Innovation Capability: 0.744.

•Digital Literacy: 0.697.

Note: * p < .05, ** p < .01, *** p < .001 Standardized coefficients are reported on paths. Error terms and residuals are shown in parentheses.

**Fig 1:**
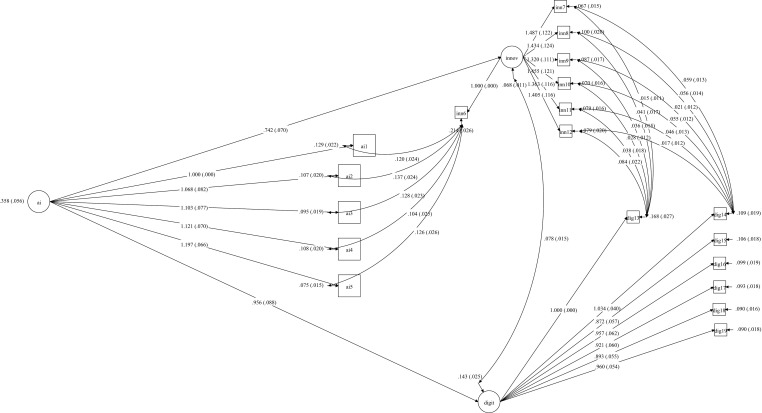
Structural Equation Model of the Relationships between AI Application, Innovation Capability, and Digital Literacy.

The structural equation model depicted in Fig 1 illustrates the complex relationships between the study’s key constructs. The measurement model demonstrates strong factor loadings across all indicators, with AI application measured through five items (AI1-AI5, λ = .858-.934), innovation capability assessed via seven indicators (INN6-INN12, λ = .744-.948), and digital literacy evaluated through seven measures (DIG13-DIG19, λ = .858-.910). All factor loadings achieved statistical significance (p < .001).

The structural paths reveal substantial standardized coefficients between AI application and both endogenous variables (βAI→Innovation = .862, βAI→Digital = .835, p < .001). The bidirectional relationship between innovation capability and digital literacy demonstrates a strong positive association (r = .791, p < .001). The model exhibits satisfactory fit indices (χ2/df = 1.704, RMSEA = .075, CFI = .963, TLI = .952, SRMR = .042), suggesting adequate representation of the empirical data.

Error terms and residuals are appropriately specified, with measurement errors for observed variables ranging from.067 to.214, indicating precise measurement. The model explains substantial variance in both innovation capability (R2 = .744) and digital literacy (R2 = .697), supporting its theoretical and practical significance.

### 4.4. Hypothesis testing results

Empirical analysis provided systematic support for all research hypotheses. The first hypothesis (H1) positing AI application’s positive influence on innovation capability received strong support through significant path coefficients (β = 0.862, p < .001). Similarly, H2 proposing AI application’s effect on digital literacy was substantiated (β = 0.835, p < .001). The third hypothesis (H3) regarding the positive association between innovation capability and digital literacy found empirical validation (β = 0.791, p < .001).

#### 4.4.1. Model modifications and comparisons.

The structural model underwent systematic refinement guided by theoretical considerations and empirical indices. Initial specification demonstrated adequate but suboptimal fit (χ²/df = 2.417, RMSEA = 0.089, CFI = 0.932, TLI = 0.918, SRMR = 0.058). Modification indices suggested substantial improvement potential through a bidirectional pathway between innovation capability and digital literacy (MI = 87.32), aligning with theoretical propositions regarding recursive relationships between cognitive capabilities and technological competencies (Cukurova, 2024).

Three alternative models were evaluated: Model A (original unidirectional specification), Model B (incorporating bidirectional pathway), and Model C (specifying AI application as a second-order construct). Nested model testing revealed significant improvement for Model B over baseline (Δχ² = 82.46, Δdf = 1, p < .001), while comparison between Models B and C demonstrated non-significant differences (Δχ² = 8.73, Δdf = 5, p = .120).

The final specification (Model B) achieved optimal balance between theoretical coherence and empirical fit, demonstrating substantial improvement across all indices (χ²/df = 1.704, RMSEA = 0.075, CFI = 0.963, TLI = 0.952, SRMR = 0.042). This specification preserves the multidimensional conceptualization of technology engagement while integrating the recursive relationship between innovation capability and digital literacy postulated in contemporary educational theories (Sharples, 2023).

### 4.5. Effects analysis

The structural model demonstrated substantial explanatory power for endogenous variables, with variance explained (R²) reaching 0.744 for innovation capability and 0.697 for digital literacy. These effect sizes, combined with significant path coefficients exceeding conventional thresholds, provide robust support for the theoretical framework’s predictive validity.

Direct effects analysis revealed standardized coefficients exceeding 0.80 for primary relationships, indicating substantial practical significance. The observed pattern of relationships demonstrated theoretical consistency while maintaining sufficient distinctiveness to support the multidimensional nature of the proposed model. The complementary effects between innovation capability and digital literacy (β = 0.791) suggest potential synergistic relationships in technology-enhanced learning environments.

## 5. Discussion

### 5.1. Analysis of research findings

The empirical evidence generated through structural equation modeling yields substantive insights into the mechanisms through which generative AI influences students’ developmental trajectories. The model’s explanatory capacity, evidenced by variance explained metrics for both innovation capability (R² = 0.744) and digital literacy (R² = 0.697), indicates that generative AI applications account for a substantial proportion of variance in these educational outcomes. The magnitude of these effects warrants systematic theoretical interpretation within contemporary educational technology paradigms.

The observed association between AI application and innovation capability (β = 0.862, p < .001) empirically substantiates the transformative potential of AI-enhanced learning environments in cultivating higher-order cognitive competencies. This finding corroborates Boelmann and Kollar’s [63] cognitive process framework, which posits that technological engagement facilitates complex problem-solving through structured cognitive mediation. The robust path coefficient suggests that AI tools function not merely as information delivery mechanisms but as cognitive scaffolds that enhance students’ capacity for innovative thinking and creative problem-solving. This empirical validation extends [64] identification of generative AI’s advantageous effects on educational outcomes while providing more precise quantification of these relationships through rigorous structural modeling techniques.

The pathway linking AI application to digital literacy (β = 0.835, p < .001) demonstrates the efficacy of technology-mediated learning in developing sophisticated digital competencies. This relationship operates through multiple mechanisms, including enhanced technological self-efficacy and cognitive engagement—a finding that substantiates Liang et al.‘s [65] theoretical propositions regarding the mediating role of these constructs in technology-enhanced learning environments. The strength of this association challenges reductionist conceptualizations of digital literacy as mere technological proficiency, instead aligning with Eshet’s [15] multidimensional framework encompassing critical evaluation, digital content creation, and ethical technology utilization.

Perhaps most theoretically significant is the bidirectional relationship between innovation capability and digital literacy (β = 0.791, p < .001), which reveals a synergistic interaction between these competencies in AI-enhanced educational contexts. This finding extends beyond [66] correlation analysis to demonstrate a reciprocal reinforcement mechanism between cognitive and technological domains. The magnitude of this association, contextualized within robust model fit indices (CFI = 0.963, TLI = 0.952), suggests that as students engage with generative AI tools, they simultaneously develop complementary competencies that mutually enhance one another—creating a virtuous developmental cycle that transcends traditional domain boundaries.

The measurement model’s psychometric properties provide robust validation for the theoretical constructs under investigation. Factor loadings across all indicators (λ = 0.744–0.948) demonstrate strong construct representation, with particularly robust manifestation in AI application indicators. This psychometric integrity enhances confidence in both the measurement model and the structural relationships derived from it, addressing methodological limitations in previous educational technology research that has often relied on less sophisticated analytical approaches.

Further examination of the relationship between generative AI and innovation capability reveals nuanced effects across its constituent dimensions. The critical thinking dimension, operationalized through analytical reasoning and evaluative judgment indicators, demonstrated particularly robust enhancement (λ = 0.744, t = 19.612, p < .001) through generative AI engagement. This finding aligns with [67] cognitive scaffolding framework, wherein AI-mediated learning environments facilitate metacognitive awareness through exposure to diverse analytical perspectives and structured interrogation of information sources. The creative problem-solving dimension exhibited the strongest factor loading (λ = 0.948, t = 78.466, p < .001), suggesting that generative AI particularly excels in fostering divergent ideation processes. This effectiveness likely stems from the algorithmic capacity to present students with novel solution pathways and unconventional problem representations that expand their conceptual search space—a mechanism elucidated in [68] computational creativity research. The adaptive learning dimension (reflected in innovation consciousness, λ = 0.943, t = 69.130, p < .001) evidenced substantial enhancement through personalized feedback mechanisms and calibrated challenge calibration. This finding extends Liang et al.‘s [65] self-efficacy mediation model by demonstrating how generative AI cultivates metacognitive regulation through iterative engagement cycles that progressively adjust to student response patterns. The differential magnitudes across these dimensions suggest that while generative AI enhances all aspects of innovation capability, its impact manifests most prominently in those dimensions involving complex pattern recognition and divergent ideation processes—competencies that align particularly well with the computational strengths of current generative algorithms. This dimensional analysis provides a more granular understanding of the specific cognitive mechanisms through which AI engagement enhances innovation capability, moving beyond aggregated effects to identify the precise pathways of cognitive enhancement.

### 5.2. Theoretical implications

The findings yield significant theoretical implications that extend across multiple dimensions of educational technology integration. First, the observed relationships provide empirical substantiation for theoretical propositions regarding the mechanisms through which technological tools influence cognitive development in educational contexts. The strong relationship between AI application and innovation capability validates Boelmann and Kollar’s [63] cognitive process framework while extending it to encompass generative AI specifically—a technology with distinct characteristics compared to traditional educational technologies.

Secondly, the findings necessitate reconsideration of how digital competencies develop within technology-enhanced learning environments. The strong bidirectional relationship between innovation capability and digital literacy challenges linear models of skill development, suggesting instead a complex recursive process through which these competencies mutually reinforce one another. This finding aligns with theoretical propositions from Cukurova [69] regarding the interplay between learning analytics and artificial intelligence, while providing empirical validation of these relationships within the specific context of generative AI applications in education.

Thirdly, the results substantiate Sharples’s [70] theoretical conceptualization of social generative AI in education, particularly regarding the integration of AI tools as cognitive amplifiers rather than mere instructional delivery mechanisms. The demonstrated relationships between AI application and student outcomes reinforce Gillani et al.’s [71] conceptualization of AI as a catalyst for enhanced learning, providing precise quantification of these effects through robust structural equation modeling. This empirical validation strengthens theoretical propositions regarding AI’s role in educational transformation while illuminating the specific pathways through which this influence operates.

Fourthly, the findings provide theoretical support for the integration of the Technology Acceptance Model [52] with Diffusion of Innovations Theory [51] in understanding technological adoption in educational contexts. The strong relationship between AI application and educational outcomes validates the theoretical proposition that perceived usefulness and relative advantage—key constructs in these frameworks—significantly influence both technology adoption and subsequent educational outcomes. This integrated theoretical approach offers a more comprehensive framework for understanding the complex processes involved in educational technology implementation than either theory alone could provide.

Finally, the research advances theoretical understanding of how generative AI specifically, as distinct from previous educational technologies, influences student development. The substantial relationships observed suggest that generative AI’s unique capabilities—including content generation, personalized feedback, and adaptive scaffolding—offer particular advantages for developing both innovation capability and digital literacy. This finding extends existing theoretical frameworks by highlighting the distinctive characteristics of generative AI that differentiate it from previous technological innovations in education.

These theoretical implications collectively suggest a need for more sophisticated conceptual frameworks that can account for the complex, recursive relationships between technological engagement and student competency development in AI-enhanced learning environments. The findings underscore the necessity of integrating insights from multiple theoretical traditions, including cognitive science, technology adoption theory, and digital literacy frameworks, to fully comprehend the transformative potential of generative AI in educational contexts.

### 5.3. Practical implications

The practical implications of this research span multiple levels of educational implementation. Firstly, the findings offer actionable insights for educators aiming to integrate generative AI tools into curricula to enhance both innovation capability and digital literacy. By utilizing AI-driven platforms that promote problem-solving and critical thinking, educators can create enriched learning environments that align with 21st-century educational objectives [9].

Secondly, the demonstrated reciprocal relationship between innovation capability and digital literacy suggests that targeted interventions in one domain can generate spillover effects in the other. For example, incorporating digital literacy training into problem-solving activities can amplify students’ innovation outcomes, providing a strategic pathway for competency development. This aligns with Nguyen et al.’s [72] findings on digital learning games and their capacity to simultaneously enhance multiple competencies. Similarly, Liang et al. [65] emphasize the importance of fostering self-efficacy and cognitive engagement as critical mediators in achieving these outcomes.

Thirdly, systematic professional development for educators is crucial to maximize the potential of generative AI in classrooms. Equipping educators with the skills to effectively utilize AI tools can ensure their alignment with pedagogical objectives, addressing challenges identified in previous studies [22]. Such initiatives should focus on enhancing educators’ own digital literacy and innovation capabilities, enabling them to model these competencies for students effectively. Nguyen et al. [73] also highlight that digital tools like ChatGPT can assist educators in designing personalized feedback mechanisms, further enhancing the learning process.

Finally, the integration of generative AI tools must address ethical and practical challenges [74]. Emphasis on the ethical use of generative AI in education underscores the need for policies that balance technological benefits with potential risks, such as data privacy concerns and over-reliance on automation. This aligns with Porayska-Pomsta’s [75] insights into human-centered AI design, advocating for systems that prioritize students’ holistic development.

### 5.4. Limitations and future research

While this investigation offers substantive empirical evidence regarding the transformative impact of generative AI on students’ competency development, several methodological limitations warrant critical consideration. The cross-sectional design, while efficient for establishing preliminary structural relationships, inherently constrains causal inference mechanisms, necessitating longitudinal investigations to delineate the temporal precedence essential for robust causal attribution. The reliance on self-reported measures, despite their established validity, introduces potential common method variance that may artificially inflate observed relationships—a limitation that future research should address through methodological triangulation incorporating behavioral assessments and performance-based measures of both innovation capability and digital literacy.

Although the primary hypothesized relationships demonstrated significant effects, several non-significant pathways emerged during model refinement that merit critical examination. Initial analyses revealed non-significant direct effects between specific AI application dimensions (particularly AI Use Frequency) and higher-order innovation processes when these dimensions were modeled separately. This finding suggests potential threshold effects in AI engagement—mere frequency of interaction may prove insufficient for cultivating sophisticated innovation capabilities without corresponding depth and diversity of application. Similarly, the anticipated moderating effect of prior technology experience on the AI-capability relationship failed to reach statistical significance (ΔR² = .02, p = .08), contradicting theoretical propositions from technology acceptance frameworks and suggesting that generative AI may function through distinct pedagogical mechanisms that transcend traditional technology integration paradigms.

The investigation confronted several potential confounding variables that warrant acknowledgment. Institutional technological infrastructure represents a critical confounding factor that may systematically influence both AI implementation opportunities and learning outcomes. Despite statistical controls for school type and geographic location, unmeasured variation in technological resources likely introduces systematic variance that remains unaccounted for in the current model. Additionally, instructor technological pedagogical knowledge constitutes a significant unmeasured variable that potentially moderates the effectiveness of AI implementation. The complex interaction between student-level variables and these contextual factors represents a critical frontier for future research examining the ecological validity of the proposed relationships.

Demographic factors introduce additional complexity that requires careful consideration. While the sampling framework ensured proportional representation across demographic categories, preliminary analyses revealed notable differences in the magnitude of AI effects across gender and grade-level subgroups, although these differences did not reach statistical significance at conventional thresholds (p > .05). This finding suggests potential developmental considerations in AI integration that merit targeted investigation through age-specific analytical models. Furthermore, the threshold approach to prior AI experience may inadequately capture the nuanced technological competencies that students bring to educational contexts, potentially masking important interaction effects.

Several methodological constraints warrant consideration when interpreting these findings. The multicollinearity observed among AI application dimensions (r = .76–.85) suggests potential construct redundancy that may inflate path coefficients despite satisfactory discriminant validity metrics. Future investigations should consider alternative measurement approaches that more distinctly isolate specific dimensions of AI engagement. Additionally, the high proportion of explained variance in both innovation capability (R² = .744) and digital literacy (R² = .697) raises questions regarding potential omitted mediating variables that may elucidate the specific mechanisms through which AI influences student outcomes. Mediation analyses incorporating cognitive engagement, self-regulated learning processes, and technology-specific self-efficacy may provide more granular understanding of these pathways.

Future research trajectories should address these limitations through methodological and conceptual advancements. Longitudinal designs incorporating at least three measurement waves would enable robust cross-lagged panel analyses to establish temporal precedence and reciprocal effects. Methodological triangulation integrating objective performance metrics, qualitative process observations, and self-report measures would mitigate common method variance concerns while providing deeper insight into the phenomenological aspects of AI-enhanced learning. Multilevel analytical frameworks that simultaneously model student, classroom, and institutional factors would more adequately capture the nested complexity of educational technology integration. Finally, experimental manipulations systematically varying specific dimensions of AI implementation would enable more precise causal inferences regarding the active ingredients of effective AI integration in educational environments.

The investigation of domain-specific effects represents another critical frontier for future research. While the current study conceptualized innovation capability and digital literacy as domain-general constructs, their manifestation likely varies across subject areas. Future investigations should examine potential disciplinary differences in how generative AI influences student competencies across STEM, humanities, and creative disciplines. Such domain-specific analyses would provide more nuanced guidance for curriculum development and pedagogical practice while advancing theoretical understanding of knowledge transfer mechanisms in technology-enhanced learning environments.

## 6. Conclusion

This study marks a significant advancement in understanding the transformative role of generative AI within educational settings, particularly in enhancing students’ innovation capability and digital literacy. By employing a robust structural equation modeling framework, the research elucidates the complex mechanisms through which generative AI influences these critical competencies. The findings affirm the hypothesized positive relationships among AI application, innovation capability, and digital literacy, thereby offering substantial theoretical and practical contributions to the field of educational technology.

The theoretical contributions of this research are multifaceted. By integrating the Innovation Diffusion Theory and the Technology Acceptance Model, the study establishes a comprehensive framework that elucidates how generative AI facilitates the development of key student competencies. This framework extends existing theories on educational technology integration by demonstrating the bidirectional relationship between innovation capability and digital literacy. Furthermore, the study builds upon the work of scholars such as Boelmann and Kollar [63] and Liang et al. [65] by empirically validating the mediating roles of self-efficacy and cognitive engagement in the relationship between AI application and capability development. These findings underscore the necessity for human-centered AI applications in education, as emphasized by Sharples [70], ensuring that AI systems align with pedagogical objectives and enhance educational outcomes.

From a practical perspective, the study offers actionable insights for educators, policymakers, and technology developers. The demonstrated positive effects of generative AI on both innovation capability and digital literacy suggest that the strategic integration of AI tools into curricula can yield substantial educational benefits. Specifically, educators can leverage AI-driven platforms to foster critical thinking, problem-solving, and ethical digital practices among students. Additionally, the findings highlight the importance of professional development programs that equip educators with the necessary skills to effectively implement AI-based tools, addressing challenges identified in previous studies [22]. Moreover, the research emphasizes the need for ethical guidelines and policies to mitigate potential risks such as data privacy concerns and over-reliance on automation, ensuring that generative AI serves as an augmentative tool rather than a replacement for human-driven educational practices.

However, this research is not without its limitations. The cross-sectional design limits the ability to draw causal inferences, necessitating future longitudinal studies to examine the temporal dynamics of AI’s impact on student competencies. Additionally, the reliance on self-reported measures may introduce response biases, underscoring the need for multimethod approaches in future research. Future investigations should also explore cultural and contextual moderators in the AI-capability development relationship, as educational environments vary significantly in terms of technological infrastructure and pedagogical practices. Expanding the scope of research to include specific AI tools, such as ChatGPT, can provide more nuanced insights into their differential impacts on various student populations, thereby informing more tailored technology integration strategies.

In conclusion, this study makes substantial contributions to both the theoretical foundations and practical applications of generative AI in education. By demonstrating the positive impacts of AI on innovation capability and digital literacy, the research provides a strategic roadmap for leveraging technology to meet the evolving demands of the educational landscape. Through continued exploration and ethical implementation, generative AI holds the potential to revolutionize education, equipping students with the essential skills required to thrive in the 21st century.

## Supporting information

S1 File Inclusivity-in-global-research-questionnaire (1). (DOCX)
